# Effect of Land Use, Seasonality, and Hydrometeorological Conditions on the K^+^ Concentration–Discharge Relationship During Different Types of Floods in Carpathian Foothills Catchments (Poland)

**DOI:** 10.1007/s11270-017-3585-0

**Published:** 2017-11-13

**Authors:** Joanna P. Siwek, Mirosław Żelazny, Janusz Siwek, Wojciech Szymański

**Affiliations:** 1 Department of Hydrology, Institute of Geography and Spatial Management, Jagiellonian University in Kraków, ul. Gronostajowa 7, 30-387 Kraków, Poland; 2 Department of Pedology and Soil Geography, Institute of Geography and Spatial Management, Jagiellonian University in Kraków, ul. Gronostajowa 7, 30-387 Kraków, Poland

**Keywords:** Potassium, Hysteresis patterns, Flood events, Hydrometeorological conditions, Land use, Foothills of middle mountains

## Abstract

The purpose of the study was to determine the role of land use, seasonality, and hydrometeorological conditions on the relationship between stream water potassium (K^+^) concentration and discharge during different types of floods—short- and long-duration rainfall floods as well as snowmelt floods on frozen and thawed soils. The research was conducted in small catchments (agricultural, woodland, mixed-use) in the Carpathian Foothills (Poland). In the woodland catchment, lower K^+^ concentrations were noted for each given specific runoff value for summer rainfall floods versus snowmelt floods (seasonal effect). In the agricultural and mixed-use catchments, the opposite was true due to their greater ability to flush K^+^ out of the soil in the summer. In the stream draining woodland catchment, higher K^+^ concentrations occurred during the rising limb than during the falling limb of the hydrograph (clockwise hysteresis) for all flood types, except for snowmelt floods with the ground not frozen. In the agricultural catchment, clockwise hystereses were produced for short- and long-duration rainfall floods caused by high-intensity, high-volume rainfall, while anticlockwise hystereses were produced for short- and long-duration rainfall floods caused by low-intensity, low-volume rainfall as well as during snowmelt floods with the soil frozen and not frozen. In the mixed-use catchment, the hysteresis direction was also affected by different lag times for water reaching stream channels from areas with different land use. K^+^ hystereses for the woodland catchment were more narrow than those for the agricultural and mixed-use catchments due to a smaller pool of K^+^ in the woodland catchment. In all streams, the widest hystereses were produced for rainfall floods preceded by a long period without rainfall.

## Introduction

Potassium (K^+^) plays an important role in the biological life in a catchment. It is a so-called biogenic ion—along with nitrogen and phosphorus (Hem 1985; Likens et al. 1994; Anderson et al. 1997; Barre et al. 2007). This assertion is confirmed by research by Neal et al. (1992) and Tetzlaff et al. (2007). Both studies note that once a forest is cut down, the release of K^+^ from soils increases permanently due to less intake by trees. K^+^ intake by plants enrich the upper layers of the soil in K^+^ thanks to a process called potassium uplift. In other words, K^+^ ions are moved up to the topsoil by plant growth (Jobbágy and Jackson 2004). Unlike in the case of nitrogen and phosphorus, the current level of knowledge on the effect of environmental factors on the K^+^ concentration in the water environment is quite insufficient, as shown by Tripler et al. (2006). One of the few papers that do provide a comprehensive analysis of K^+^ circulation in catchments is that of Likens et al. (1994), which is based on years of hydrochemical research at Hubbard Brook experimental catchment in New Hampshire (USA). This paper reviews the main causes of long-term and seasonal changes in K^+^ concentrations in precipitation water and stream water.

Information on rates of change in the K^+^ concentration during flood events comes primarily from papers on the so-called main ions, which include K^+^ (Edwards 1973; Foster 1978; Hill 1993; Anderson et al. 1997; Sandén et al. 1997; Evans and Davies 1998; Siwek et al. 2011). Most of these papers note that K^+^ reacts differently to increased discharge than do other main ions. K^+^ concentrations tend to increase during the rising limb of a flood event (Edwards 1973; Foster 1978; Evans and Davies 1998; Siwek et al. 2011) or remain the same (Hill 1993; Salmon et al. 2001). On the other hand, most main ions tend to decline in concentration under similar conditions. Some of the above papers also note a weaker relationship between K^+^ and discharge—relative to that for other main ions (Anderson et al. 1997; Siwek et al. 2011). This difference is often attributed to the complex geochemical properties of K^+^ (Evans et al. 1996). On the one hand, K^+^ ions are easily adsorbed by clay minerals and organic matter, which reduce their concentration in stream water (Likens et al. 1994; Barré et al. 2007, 2008). On the other hand, potassium salts such as sylvite, kainite, and polyhalite tend to readily dissolve in water, which increases the K^+^ concentration in water (Hem 1985).

The weak relationship between K^+^ and discharge is also linked with the origin of K^+^ from a variety of sources. In natural catchments (i.e., woodland catchments), K^+^ in stream water may originate from the leaching of bedrock (Caissie et al. 1996), ion exchange (Williams et al. 2001), erosion and soil flushing (Edwards 1973; Evans and Davies 1998; Holloway and Dahlgren 2001), decomposition of organic matter (Stottlemyer 2001), and dry and wet atmospheric deposition (Likens et al. 1994). In woodland catchments, K^+^ can be washed out of or washed from the surface of needles and bark (Likens et al. 1994; Evans and Davis 1998; Rothe et al. 2002; Małek and Astel 2008; Likens 2013). Another key reservoir of K^+^ consists of 2:1 clay minerals occurred in the upper horizons of the soil that adsorb K^+^ ions (Barré et al. 2007, 2008). Clay minerals are an important part of stream channel sediments. K^+^ concentrations in stream water increase rapidly in the early stages of many flood events due to the erosion of clay minerals (Sandén et al. 1997). According to laboratory experiments by Hill (1993), initial K^+^ concentrations in storm runoff increased by 19–28% during a contact time of 20–60 min with swamp substrates. Koerselman et al. (1993) observed increased loss of K^+^ in soils that have been frozen for 1 week, which they attributed to the release of K^+^ from decaying microorganisms. Human impact in a stream catchment also leads to an increased potassium load in stream water in the form of wastewater (Neal et al. 2000) and in the form of increased organic and inorganic fertilizer content (Holz 2010). K^+^ reaches stream channels from a wide variety of sources and as part of various components of discharge including groundwater, throughflow, and runoff. Evans and Davies (1998) believe that K^+^ concentrations in stream water are determined by the sequence of runoff components and the relative content of K^+^ in each given component. The relationship between the K^+^ concentration and discharge often takes the form of a hysteresis, which shows that the concentration of K^+^ at a given discharge is different during the rising limb from that during the falling limb (Foster 1978; Evans and Davies 1998; Holz 2010). In effect, the K^+^ concentration versus discharge relationship can be used to make inferences about key hydrologic processes occurring in a studied catchment (Anderson et al. 1997; Evans and Davies 1998; Siwek et al. 2011, 2013a).

There still exists a large knowledge gap in the area of factors that impact K^+^ concentrations in stream water during flood events. This gap is relevant, as the largest loads of K^+^ enter river water during flood events. The impact of environmental factors such as flood type on the loss of K^+^ from catchments is important—and must be considered when creating a schedule of potassium fertilizer use in agricultural areas. An effective schedule will reduce K^+^ loss from the soil and enhance plant growth. Knowledge on human impact in the form of the types of land use may be useful in spatial planning (i.e., housing development) in areas threatened by the eutrophication of water. The determination of the role of any natural and anthropogenic factors in changes in the K^+^ concentration during flood events requires the use of detailed analysis (e.g., high sampling frequency) in different types of catchments during various types of flood events. Research has traditionally been conducted in single catchments—in most cases woodland catchments (Sandén et al. 1997; Stottlemyer 2001; Williams et al. 2001) experiencing one type of flood event, usually a storm-driven flood (Foster 1978; Hill 1993; Sandén et al. 1997). The purpose of this paper is to answer the following three questions:

1. What is the role of hydrometeorological conditions such as (i) the amount and intensity of precipitation, (ii) changes in discharge, and (iii) the condition of the soils (moisture levels, degree of freezing) in the relationship between stream water K^+^ concentration and discharge during a flood event?
2. Do changes in the K^+^ concentration in stream water depend on flood type and seasonality?
3. What role in the K^+^ concentration in stream water during flood events do catchment land use and catchment size play?

The paper discusses key flood types identified in the Carpathian Foothills, which were classified using the factors that trigger them as well as based on environmental conditions affecting each given flood event: (i) rainfall floods and (ii) snowmelt floods and floods caused by rainfall on snow. Floods were also distinguished as (i_a_) storm floods lasting several hours, and (i_b_) long rainfall floods caused by stationary atmospheric fronts producing precipitation over the course of several days, but in some cases lasting more than 10 days. In addition, snowmelt floods and floods caused by rainfall on snow were separated into two categories: (ii_a_) with the soil frozen and (ii_b_) with the soil not frozen.

## Study Area

**Fig. 1:**
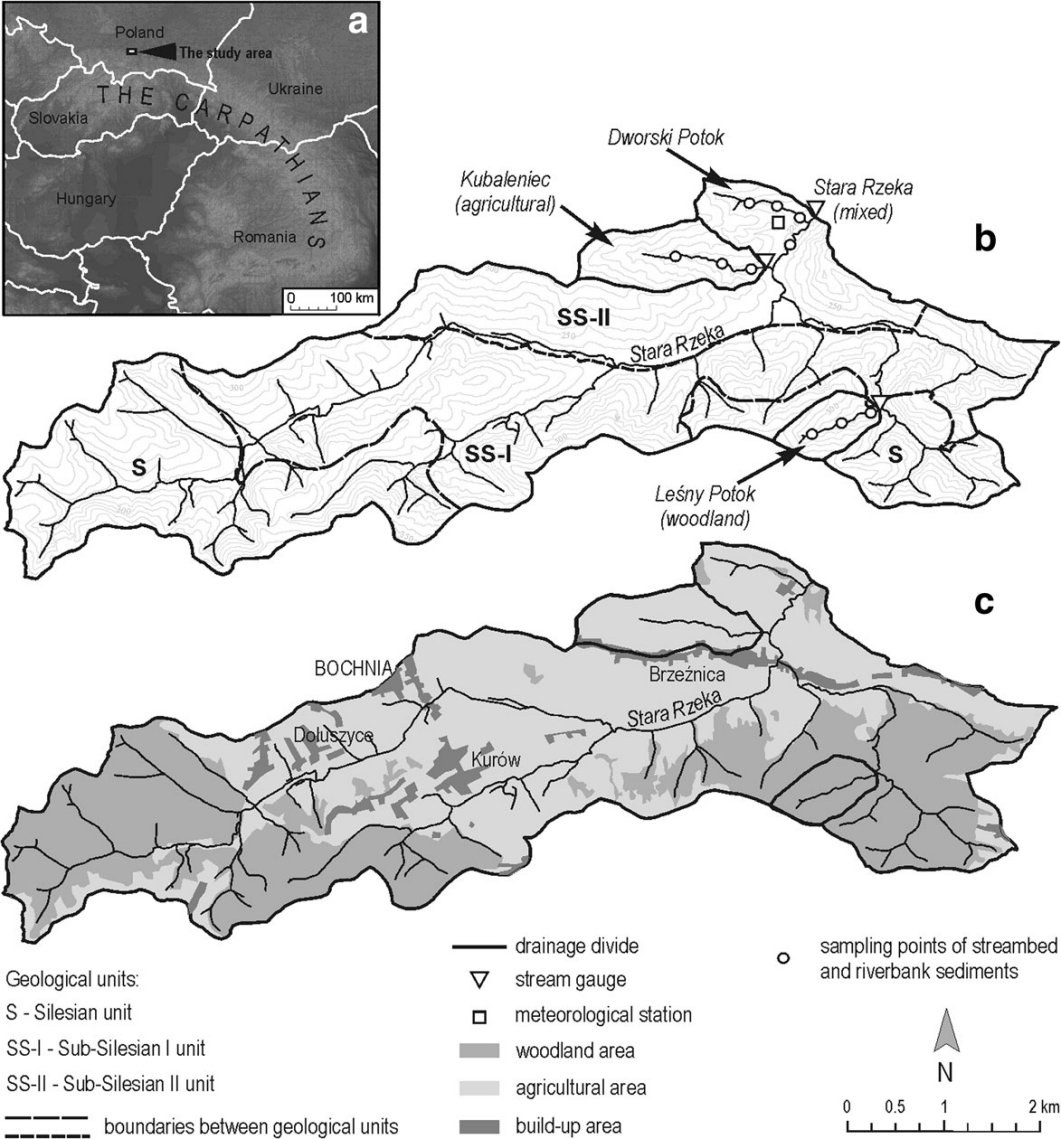
Location of the study area in the Carpathians (**a**). Hypsometry and geology of the Stara Rzeka catchment (**b**). Land use of the Stara Rzeka catchment (**c**)

The study area consists of the northern edge of the Carpathian Foothills in southern Poland and includes the Stara Rzeka catchment characterized by mixed land use. The study area was divided into two sub-catchments—the Leśny Potok woodland sub-catchment and the Kubaleniec agricultural sub-catchment (Fig. 1). The elevation of the Stara Rzeka catchment ranges from 217 to 362 m a.s.l.; mean hillslope is 7.65° (Siwek et al. 2013b).The dominant type of relief are low and medium hills (Święchowicz 2002). The geology of the study area includes two distinct units known as the Silesian and the Sub-Silesian (I and II) units, which consist of Cretaceous and Tertiary (Miocene) flysch formations. The Silesian unit consists of mostly sandstone and shale, while the Sub-Silesian unit I consists of sandstone, claystone, shale, clay, and conglomerates. The Sub-Silesian unit II consists of claystone, marl clay, gypsum, sandstone, and salt (Olewicz 1973). The studied catchment features a thick mantle of loess (called also loess-like deposits) exceeding 10 m in thickness in some sites. The loess is a parent material of Luvisols and Retisols (IUSS Working Group WRB 2015), which are dominant soil units in the study area (Skiba 1992; Skiba et al. 1998; Szymański et al. 2011; Szymański and Skiba 2013). The soils are characterized by a permeable surface and eluvial horizons, which are underlain by a hardly permeable illuvial (argillic, fragipan) horizon with clearly higher content of clay fraction than upper part of soil profile. Improper farming such as plowing along a slope has led to some erosion of the eluvial horizon, exposing illuvial horizon on the surface (Skiba et al. 1998).

The land use structure in the Stara Rzeka catchment (surface area, 22.22 km^2^) is as follows: 42% woodland, 36% arable land, and 15% meadows and pastures. Local villages, such as Brzeźnica, Kurów, and Dołuszyce (Fig. 1), discharge wastewater that diminishes the quality of the local groundwater and surface water. Local communities do possess water mains, wells, and springs. However, there is no community sewer system, which leads to 90% of local wastewater being left untreated. Most household and farm wastewater is released into roadside ditches that eventually channel water into local streams. Local farmland is routinely covered with solid manure in order to make the soil more fertile. Liquid manure is used to make flat-bottomed valleys more fertile. Pure potassium fertilizer use in the Małopolska region stands at about 19.7 kg ha^−1^ year^−1^, while the national average in Poland is 29.4 kg ha^−1^ year^−1^ (Central Statistical Office 2011). The average for the European Union is 40 kg ha^−1^ year^−1^ (Barré et al. 2007).

The Kubaleniec sub-catchment (1.03 km^2^) is a typical agricultural catchment featuring hilly relief (low hills) located atop the Sub-Silesian II geologic unit. The elevation of the catchment ranges from 223 to 296 m a.s.l.; mean hillslope is 6.22° (Siwek et al. 2013b). The land use structure is as follows: 69% arable land, 20% meadows and pastures, and 0.5% woodland. The area is dominated by farms featuring long and narrow plots (Święchowicz 2002). The village of Brzeźnica is located along the boundary of the catchment and its effluent contributes substantially to stream water pollution in the region, as household and farm wastewater is discharged directly into the flat-bottomed Kubaleniec Valley. The Kubaleniec catchment exhibits a prevalence of Luvisols and Retisols on slopes and summits of hills. Gleysols exhibiting clear evidence of anaerobic conditions due to the shallow occurrence of groundwater are present along the stream.

The Leśny Potok sub-catchment (0.48 km^2^) is located atop the Silesian geologic unit. The elevation of the catchment ranges from 257 to 342 m a.s.l (medium hills); mean hillslope is 9.99° (Siwek et al. 2013b). More than 99% of the area is covered with forest between 40 and 80 years old, consisting mostly of beech (*Fagus sylvatica* L.), fir (*Abies alba* Mill.), and complexes closely genetically linked to mixed *Pino-Quercetum* forests. The area is characterized by a flat valley bottom populated with young alder (*Alnus incana* L.). The catchment features many steep V-shaped valleys forming deep-cutting badlands and this is the main reason that this area is not inhabited by people. Luvisols and Retisols found on slopes and summits of hills as well as Gleysols found across the bottoms of valleys prevail in the Leśny Potok catchment. The main difference between soils occurring in the Leśny Potok catchment and those in the Kubaleniec catchment is that Luvisols and Retisols are more eroded in the latter catchment due to plowing along slopes.

## Methods

### Field Measurements and Laboratory Analysis

**Fig. 2:**
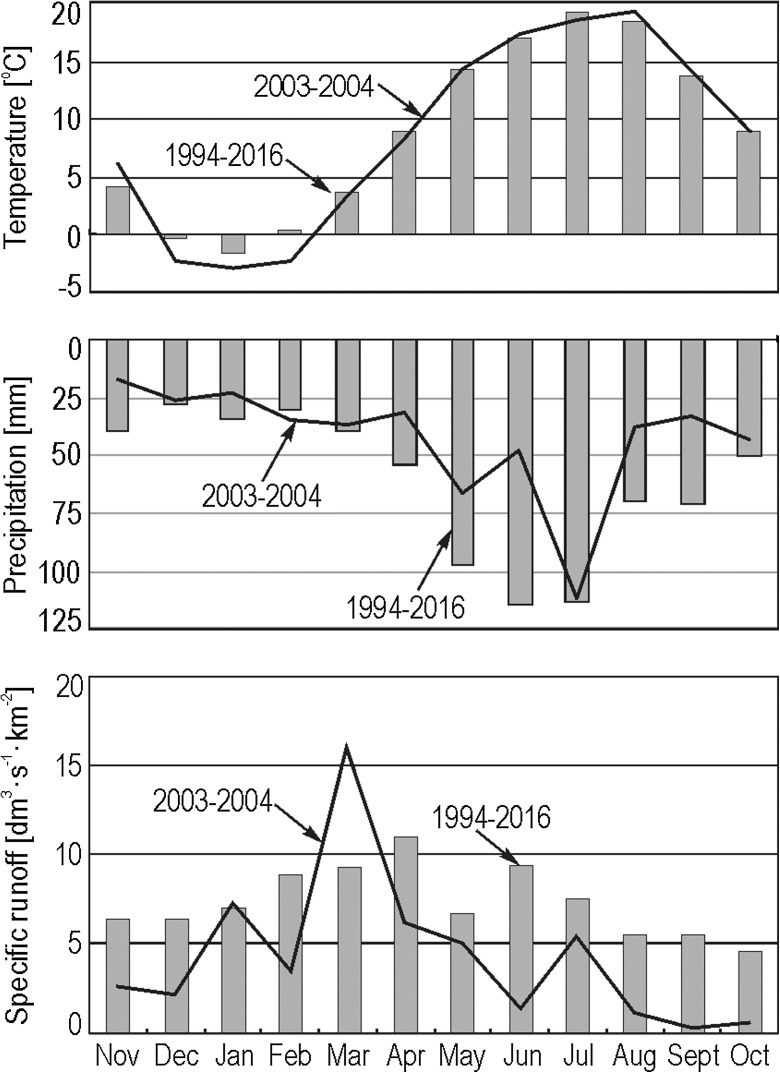
Average monthly air temperatures, precipitation and specific runoff values in the Stara Rzeka catchment during the study period (2003–2004 hydrological years) and multi-year period (1993–2016)

Research work was conducted in the period 2002–2004. Water samples were collected at a number of gauging sites in each sub-catchment—Leśny Potok and Kubaleniec (Fig. 1). A total of 29 flood events were studied—of which seven were triggered by prolonged rainfall. Another seven floods were triggered by high-intensity storm rainfall and the remaining 15 floods were snowmelt floods. The 2003 and 2004 hydrological years, in which most of the floods were sampled, were characterized by a slightly lower average annual air temperature (8.5 °C) than a multi-year average (8.9 °C in 1993–2016 hydrological years), lower average annual precipitation (522.5 mm in 2003–2004, 720 mm in 1993–2016), and lower average annual specific runoff in the Stara Rzeka catchment (4.4 dm^3^ s^−1^ km^−2^ in 2003–2004, 7.3 dm^3^ s^−1^ km^−2^ in 1993–2016). The distribution of average monthly temperatures, precipitation, and specific runoff of Stara Rzeka in 2003–2004 hydrological years generally resembled that of the analyzed multi-year period (Fig. 2). The details on the hydrometeorological conditions present at the time of each flood are available in Table 1 and in Siwek et al. (2011, 2013b). Water samples were obtained manually at time intervals ranging from minutes to hours, depending on the rate of flow at a given point in time and the duration of the flood event. Each field observation included visual inspection of overland flow during each flood event. Water samples from overland flow were collected manually during selected flood events at sites not equipped with instruments. The sampling site distribution represented different types of land use—arable land, meadows, and forest paths. Water samples were filtered through SARTORIUS (0.45 μm) filters. K^+^ concentrations were measured using a JENWAY PFP 7 flame photometer.

**Table 1:** Hydrometeorological characteristics of studied floods

Catchment	Time	Flood type	The maximum specific runoff [dm^3^ s^−1^ km^−2^]	Amount of precipitation [mm]
Woodland	January 14–16, 2003	S_f_1	7.3	0.5
	March 9–15, 2003	S_f_2	156.9	25.5
	July 10–11, 2003	R_s_1	16.7	8.1
	January 12–15, 2004	S_u_1	4.6	6.4
	June 20–21, 2004	R_s_2	13.8	6.9
	July 27–31, 2004	R_p_1	402.1	110.6
Agricultural	July 15–16, 2002	R_s_1	0.6	3.8
	July 16–17, 2002	R_s_2	235.2	40.4
	August 13–21, 2002	R_p_1	50.0	43.4
	October 17–November 7, 2002	R_p_2	79.3	60.5
	January 14–16, 2003	S_f_1	10.5	0.5
	March 9–15, 2003	S_f_2	228.8	25.5
	January 12–15, 2004	S_u_1	10.5	6.4
	February 1–9, 2004	S_u_2	12.8	17.0
	March 10–19, 2004	S_u_3	77.9	0.2
	March 25–30, 2004	S_u_4	80.4	29.5
	July 27–August 1, 2004	R_p_3	103.1	110.6
Mixed	July 15–16, 2002	R_s_1	10.0	3.8
	July 16–17, 2002	R_s_2	184.4	40.4
	August 13–21, 2002	R_p_1	19.4	43.4
	October 17–November 9, 2002	R_p_2	64.8	62.0
	January 14–16, 2003	S_f_1	8.5	0.5
	March 9–15, 2003	S_f_2	334.3	25.5
	January 12–16, 2004	S_u_1	8.5	6.4
	February 1–10, 2004	S_u_2	17.7	17.0
	March 10–20, 2004	S_u_3	45.6	2.6
	March 25–30, 2004	S_u_4	47.0	29.5
	July 23–25, 2004	R_s_3	13.0	19.8
	July 26–August 1, 2004	R_p_3	200.7	110.6

*R*_*s*_ storm rainfall floods, *R*_*p*_ prolonged rainfall floods, *S*_*f*_ snowmelt floods with the soil frozen, *S*_*u*_ snowmelt floods with the soil unfrozen, *1*,*2*,*3*… number of consecutive floods

Discharge was determined at gauging sites continuously measuring water levels (float gauge, until May 2003) and in 10-min time intervals (pressure-type water level sensors, after May 2003). Discharge was calculated using rating curves produced using a procedure by Wanielista et al. (1997) and Dingman (2002). The rating curve was extrapolated for Kubaleniec gauge data, but the range of extrapolation did not exceed 10% of measured water stages. Meteorological data were obtained from a station located in the Stara Rzeka River catchment (Fig. 1).

Samples of streambed and riverbank (both sides) sediments were collected in the upper, middle, and lower parts of the studied streams. In addition, the samples were collected also from one of the tributaries of the Stara Rzeka–Dworski Potok, which drain agricultural catchment (Fig. 1). The samples were air dried, crushed using a wooden rolling pin, and screened through a 2-mm sieve. Physical properties and content of exchangeable K^+^ were determined in the fine earth material (fraction < 2 mm). Particle-size distribution was determined using a sieving (for sand fraction) and a hydrometer method (for silt and clay fractions) (Gee and Bauder 1986). Concentration of exchangeable K^+^ were measured by flame atomic absorption spectrometry (FAAS) after extraction with 1 M ammonium acetate at pH = 7 (Sumner and Miller 1996).

### Statistical Analysis

**Fig. 3:**
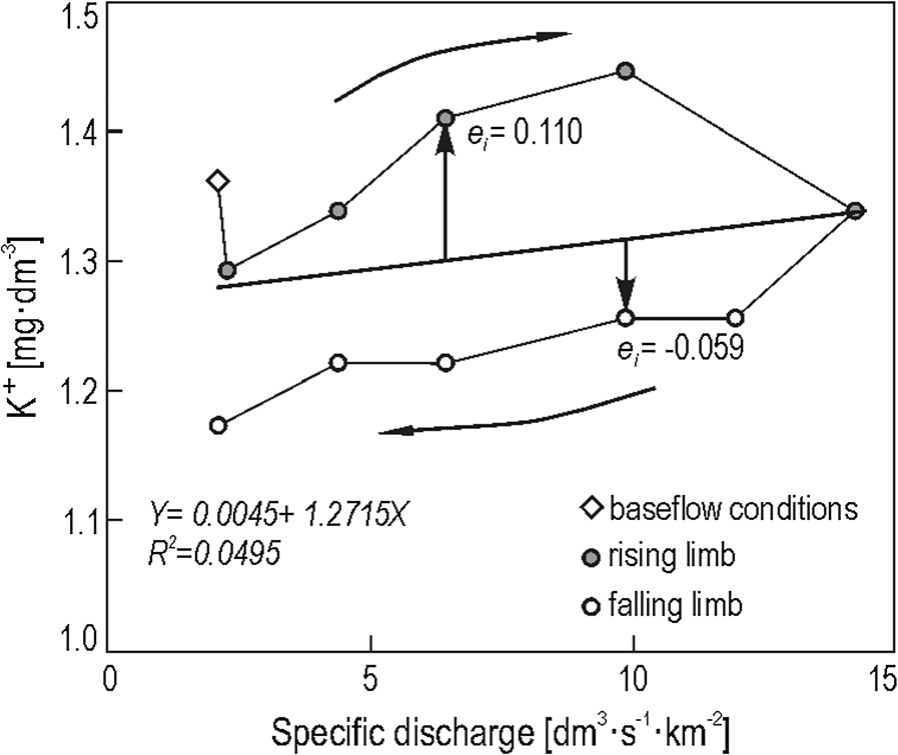
Scheme of determining of regression residuals based on K^+^ concentration changes of the Leśny Potok (woodland) stream water during summer storm event (June 20–21, 2004)

The relationship between the K^+^ concentration and discharge was determined using the linear regression equation *Ŷ* = *a* + *bX*, where *Ŷ* stands for estimated *Y* values (K^+^ concentration mg dm^−3^), *a* the intercept, *b* the slope of the regression line, and *X* the independent variable (discharge dm^3^ s^−1^). The strength of the relationship between the K^+^ concentration and discharge was determined via the coefficient of determination *R* ^2^, which shows the percentage of the variation in the dependent variable that can be explained by variation in the independent variable (Shaw and Wheeler 1997). The direction and width of hystereses were determined using standard graphing methods as well as a novel method proposed by Siwek et al. (2013b) that appears to produce more objective results. This new method is based on an analysis of average regression residuals K^+^ versus discharge for the rising limb *ē*_r_ and the falling limb *ē*_f_ of a flood event (Fig. 3). Clockwise hystereses tend to yield average residuals in the rising limb that exceed those in the falling limb (*ē*_r_ > *ē*_f_). The opposite is true in the case of anticlockwise hystereses (*ē*_r_ < *ē*_f_). The gap between average residuals calculated for the rising limb and the falling limb (|*ē*_r_| + |*ē*_f_|) indicates the size of the hysteresis loop. The calculations were done using Statistica 10.0.

## Results

**Fig. 4:**
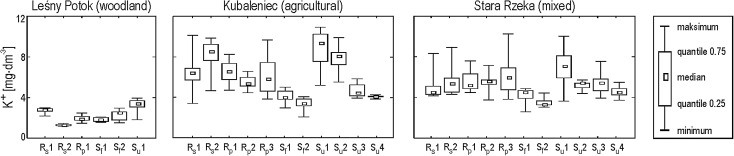
Statistical characteristics of K^+^ concentration in the three studied streams during different types of floods (R_s_, storm rainfall floods; R_p_, prolonged rainfall floods; S_f_, snowmelt floods with the soil frozen; S_u_, snowmelt floods with the soil unfrozen; 1,2,3…, number of consecutive floods)

K^+^ concentrations in the stream water were markedly lower in the woodland catchment versus the agricultural and mixed-use catchments for all flood types (Fig. 4). Furthermore, K^+^ concentrations were clearly lower in the period preceding flood events in the studied woodland catchment (Table 2). Changes in the K^+^ concentration were also smaller in the stream draining woodland catchment relative to other catchment types during the studied flood events (Fig. 4). The concentration of K^+^ decreased in the course of each subsequent snowmelt flood event (S_f_1–S_f_2, S_u_1–S_u_4, see Fig. 4) in the agricultural and mixed-use catchments. In the woodland catchment, the opposite was true. The K^+^ concentration was actually higher during the second of two subsequent snowmelt flood events.

**Table 2:** K^+^ concentration in an event water and in a baseflow [mg dm^−3^]

Catchment	Time	Flood type	Baseflow	Event water		
				Median	Mean	Maximum
Woodland	January 14–16, 2003	S_f_1	1.6	1.9	1.9	2.1
	March 9–15, 2003	S_f_2	2.6	2.6	2.3	3.0
	July 10–11, 2003	R_s_1	2.3	2.8	2.7	3.0
	January 12–15, 2004	S_u_1	1.9	3.5	3.5	4.0
	June 20–21, 2004	R_s_2	1.4	1.3	1.3	1.4
	July 27–31, 2004	R_p_1	1.6	1.9	1.9	2.5
Agricultural	July 15–16, 2002	R_s_1	3.4	6.9	6.5	10.1
	July 16–17, 2002	R_s_2	4.6	8.6	8.6	9.9
	August 13–21, 2002	R_p_1	7.3	6.4	6.3	8.3
	October 17–November 7, 2002	R_p_2	4.5	5.6	5.6	6.6
	January 14–16, 2003	S_f_1	2.9	4.2	4.0	5.0
	March 9–15, 2003	S_f_2	2.0	3.5	3.4	4.1
	January 12–15, 2004	S_u_1	5.2	9.2	9.5	10.9
	February 1–9, 2004	S_u_2	5.5	8.1	8.3	9.9
	March 10–19, 2004	S_u_3	3.9	4.7	4.5	5.8
	March 25–30, 2004	S_u_4	4.0	4.1	4.1	4.2
	July 27–August 1, 2004	R_p_3	3.8	6.3	5.9	9.7
Mixed	July 15–16, 2002	R_s_1	4.4	4.6	5.0	8.3
	July 16–17, 2002	R_s_2	4.3	5.4	5.7	8.9
	August 13–21, 2002	R_p_1	4.9	5.3	5.6	7.6
	October 17–November 9, 2002	R_p_2	3.8	5.6	5.7	7.2
	January 14–16, 2003	S_f_1	2.6	4.5	4.4	4.9
	March 9–15, 2003	S_f_2	3.3	3.3	3.5	4.4
	January 12–16, 2004	S_u_1	3.6	7.5	7.5	10.0
	February 1–10, 2004	S_u_2	4.4	5.4	5.3	5.7
	March 10–20, 2004	S_u_3	4.8	5.5	5.3	7.6
	March 25–30, 2004	S_u_4	5.5	4.5	4.4	4.9
	July 23–25, 2004	R_s_3	6.4	7.8	8.2	10.2
	July 26–August 1, 2004	R_p_3	6.4	5.8	6.0	10.2

*R*_*s*_ storm rainfall floods, *R*_*p*_ prolonged rainfall floods, *S*_*f*_ snowmelt floods with the soil frozen, *S*_*u*_ snowmelt floods with the soil unfrozen, *1*,*2*,*3*… number of consecutive floods

**Fig. 5:**
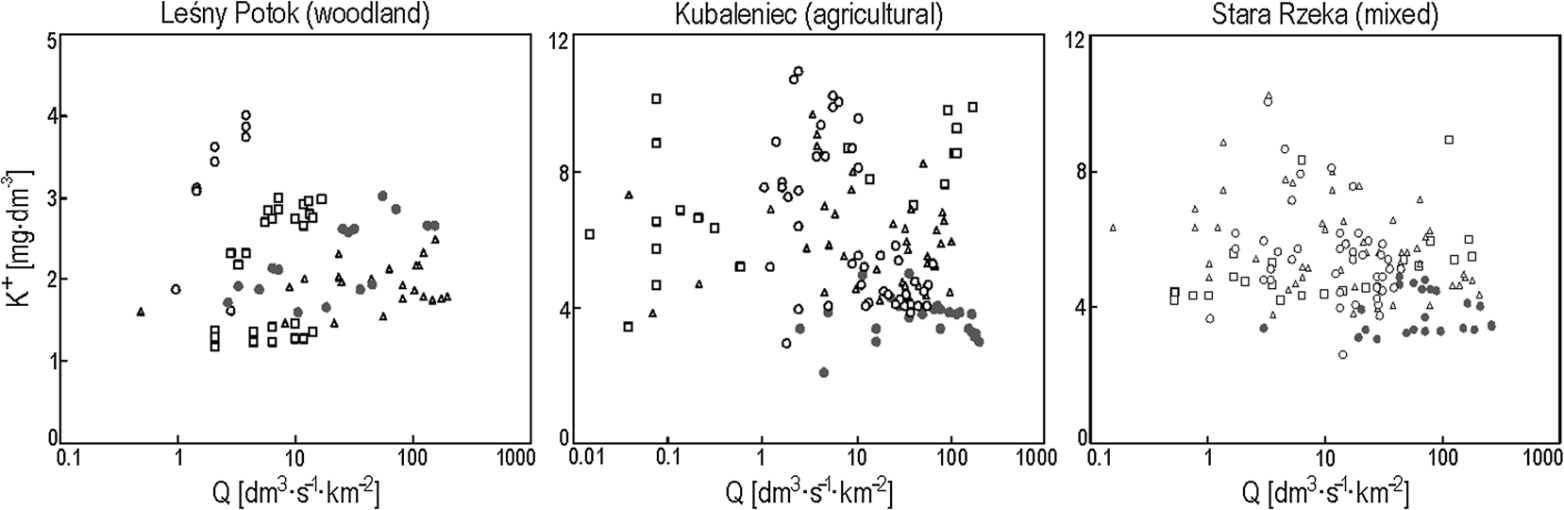
K^+^ concentration versus specific runoff Q in the three streams with respect to particular types of floods (square, intensive storm events; triangle, prolonged rainfall events; filled circle, snowmelt events with the soil frozen; empty circle, snowmelt events with the soil unfrozen)

In the woodland catchment, the concentration of K^+^ was higher at each given specific runoff value during snowmelt events with the ground unfrozen versus summer rainfall floods (storms and long-lasting rainfall) and snowmelt floods with the ground frozen. The opposite was true in both agricultural and mixed-use catchments, as the concentration of K^+^ was higher during summer rainfall floods versus mid-winter and spring floods driven by the melting of snow. Lower K^+^ concentrations were noted during snowmelt floods with the ground frozen versus snowmelt floods with the ground not frozen (Fig. 5).

**Fig. 6:**
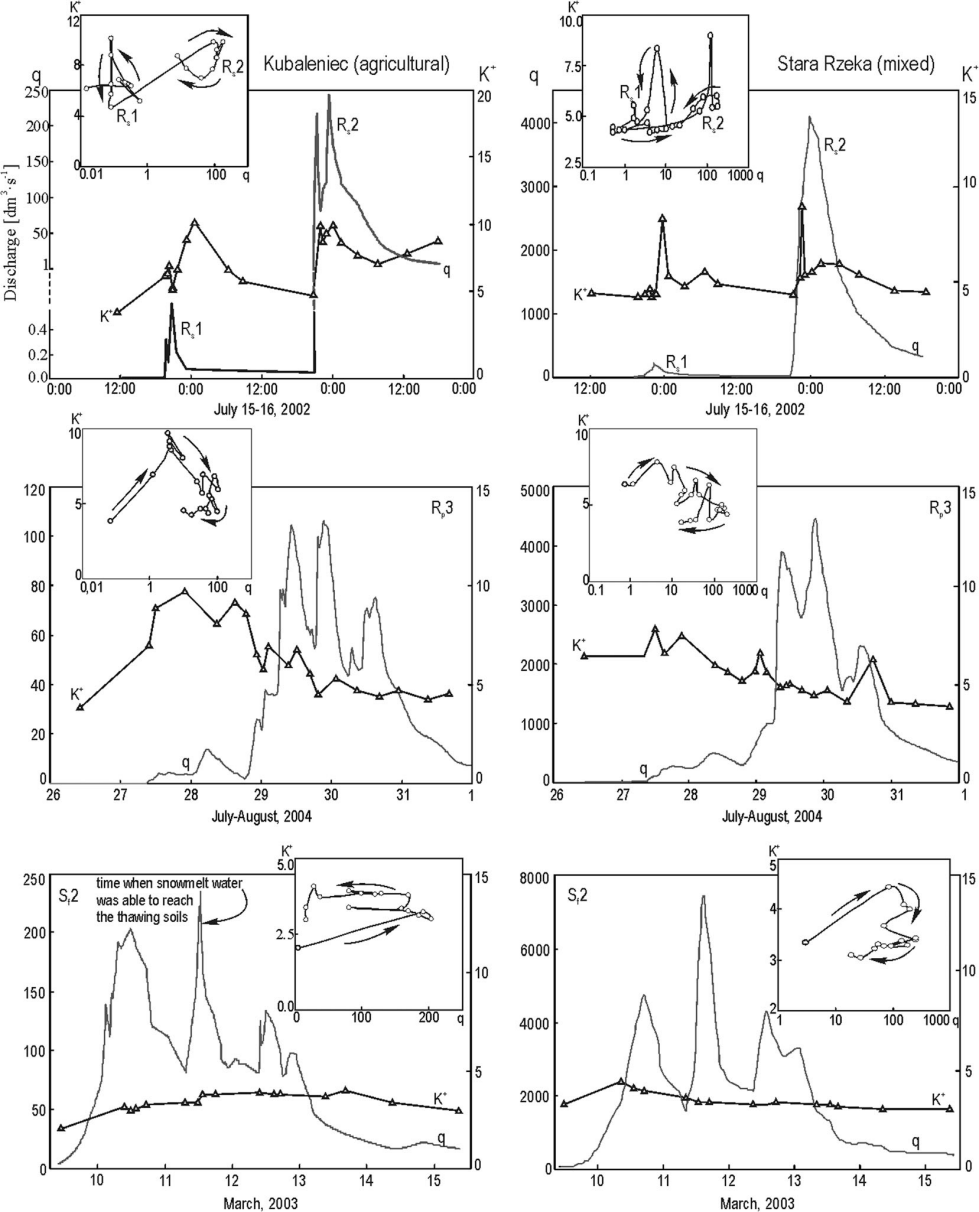
Changes of stream water K^+^ concentration of Kubaleniec and Stara Rzeka during floods of different types: storm rainfall floods (R_s_), prolonged rainfall floods (R_p_), and snowmelt floods with the soil frozen (S_f_)

The concentration of K^+^ increased in all the studied streams at the beginning of each studied flood event, as discharge began to increase. Afterwards, concentration patterns varied, with concentration sometimes decreasing and sometimes increasing or remaining the same (Fig. 6). Such variations in the concentration of K^+^ in the course of selected flood events produced a weak or statistically insignificant relationship between K^+^ concentration and discharge, as expressed by low coefficients of regression *R* ^2^ (Table 3).

**Table 3:** Direction of K^+^ hysteresis loops based on residuals from regression analysis

Catchment	Time	Flood type	Average values of residuals from regression analysis [mg dm^−3^]		*R* ^2^	*p*	Hysteresis direction
			Rising limb	Falling limb			
Woodland	January 14–16, 2003	S_f_1	0.012	− 0.025	0.814	0.014	Clockwise
	March 9–15, 2003	S_f_2	0.180	− 0.103	0.232	0.134	Clockwise
	July 10–11, 2003	R_s_1	0.137	− 0.082	0.456	0.006	Clockwise
	January 12–15, 2004	S_u_1	− 0.149	0.186	0.652	0.009	Counterclockwise
	June 20–21, 2004	R_s_2	0.073	− 0.077	0.050	0.050	Clockwise
	July 27–31, 2004	R_p_1	0.037	− 0.022	0.049	0.308	Clockwise
Agricultural	July 15–16, 2002	R_s_1	− 0.225	0.869	0.041	0.574	Counterclockwise
	July 16–17, 2002	R_s_2	0.458	0.124	0.489	0.024	Clockwise
	August 13–21, 2002	R_p_1	− 0.391	0.364	0.276	0.119	Counterclockwise
	October 17–November 7, 2002	R_p_2	− 0.229	0.448	0.598	0.015	Counterclockwise
	January 14–16, 2003	S_f_1	− 0.231	0.581	0.062	0.462	Counterclockwise
	March 9–15, 2003	S_f_2	− 0.274	0.177	0.007	0.760	Counterclockwise
	January 12–15, 2004	S_u_1	− 0.423	1.042	0.204	0.164	Counterclockwise
	February 1–9, 2004	S_u_2	0.136	− 0.068	0.000	0.964	Clockwise
	March 10–19, 2004	S_u_3	− 0.305	0.279	0.008	0.707	Counterclockwise
	March 25–30, 2004	S_u_4	0.022	− 0.011	0.230	0.336	Clockwise
	July 27–August 1, 2004	R_p_3	0.821	− 0.423	0.162	0.079	Clockwise
Mixed	July 15–16, 2002	R_s_1	− 0.711	0.860	0.050	0.484	Counterclockwise
	July 16–17, 2002	R_s_2	0.274	− 0.141	0.254	0.137	Clockwise
	August 13–21, 2002	R_p_1	0.456	− 0.381	0.730	0.001	Clockwise
	October 17–November 9, 2002	R_p_2	0.231	− 0.111	0.592	0.015	Clockwise
	January 14–16, 2003	S_f_1	− 0.009	0.285	0.411	0.034	Counterclockwise
	March 9–15, 2003	S_f_2	0.153	− 0.086	0.083	0.296	Clockwise
	January 12–16, 2004	S_u_1	0.350	− 0.438	0.264	0.158	Clockwise
	February 1–10, 2004	S_u_2	− 0.182	0.091	0.009	0.811	Counterclockwise
	March 10–20, 2004	S_u_3	0.467	− 0.536	0.044	0.374	Clockwise
	March 25–30, 2004	S_u_4	0.399	− 0.399	0.131	0.481	Clockwise
	July 23–25, 2004	R_s_3	− 0.368	0.520	0.046	0.645	Counterclockwise
	July 26–August 1, 2004	R_p_3	0.315	− 0.325	0.229	0.024	Clockwise

*R*_*s*_ storm rainfall floods, *R*_*p*_ prolonged rainfall floods, *S*_*f*_ snowmelt floods with the soil frozen, *S*_*u*_ snowmelt floods with the soil unfrozen, *1*,*2*,*3*… number of consecutive floods, *R* ^2^ coefficient of determination, *p* statistical significance)

**Fig. 7:**
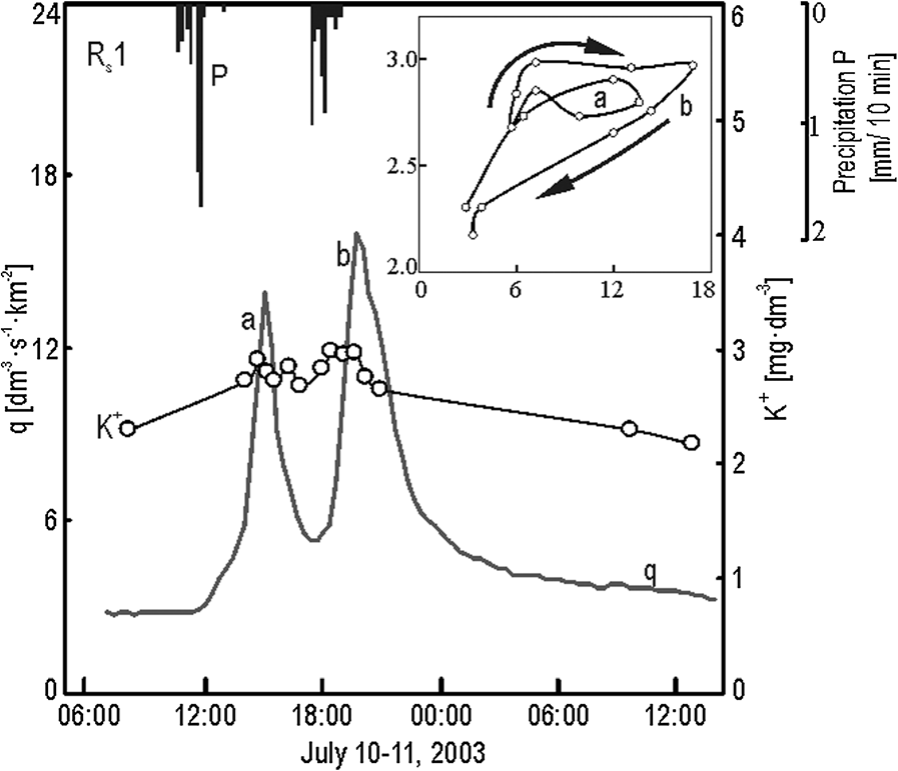
Changes of stream water K^+^ concentration of Leśny Potok during summer storm event

**Fig. 8:**
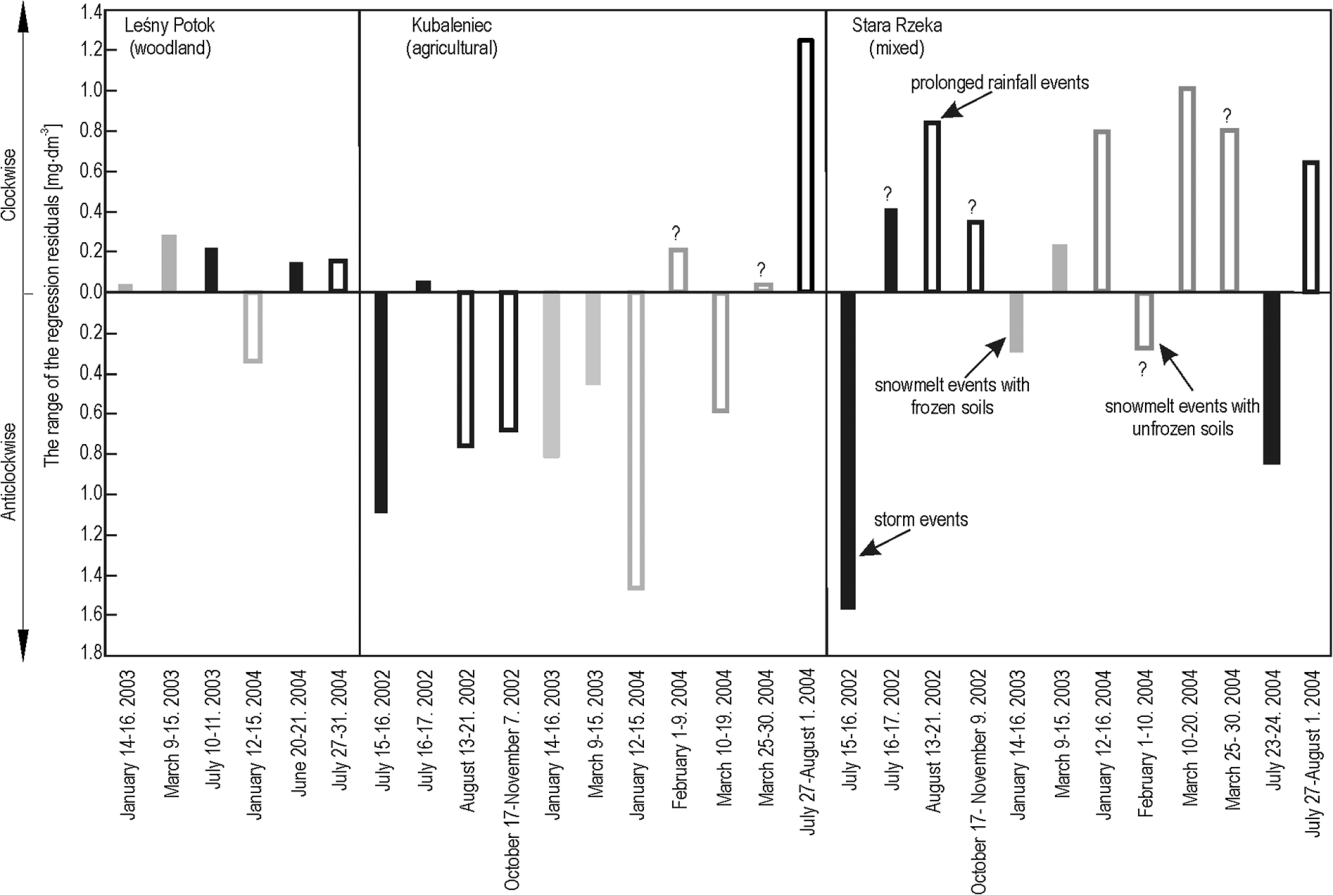
Range between average regression residuals of the rising and the falling limbs of the hydrographs (|*ē*_r_| + |*ē*_f_|) in the three studied streams during different types of floods. Question mark indicates that changes in the concentration of K^+^ were so complex that an unambiguous classification as clockwise or anticlockwise hystereses based on graphical analysis was not possible, despite this being the classification suggested by an analysis of regression residuals

The hysteresis effect was noted for K^+^ concentrations versus discharge during flood events in each studied stream. This effect can be summarized as a change in the K^+^ concentration from the rising limb of a flood to its falling limb. In the stream draining the woodland catchment, higher K^+^ concentrations were measured during the rising limb versus the falling limb for all flood types—except in the case of snowmelt floods with the soil not frozen (Fig. 7, see also Fig. 3). K^+^ hysteresis noted for the woodland catchment were visibly narrower compared with those for the agricultural and mixed-use catchments. This narrow pattern is illustrated via smaller differences between average regression residuals for the rising limb and the falling limb (Fig. 8).

Anticlockwise hysteresis was predominant in the agricultural catchment—seven out of 11 cases. This was the case for all snowmelt floods with the ground frozen (S_f_1, S_f_2; see Fig. 6), long-lasting rainfall floods (R_p_1 = 9 days; R_p_2 = 22 days) caused by high precipitation (R_p_1 = 43.4 mm; R_p_2 = 60.5 mm) characterized by low mean intensity (R_p_1 = 0.25 mm h^−1^; R_p_2 = 0.15 mm h^−1^). This was also the case for a small storm-driven flood preceded by a relatively long (11 days) period without rainfall (R_s_1; Fig. 6). Anticlockwise K^+^ hysteresis in the stream draining the agricultural catchment was also during two out of four snowmelt flood events with the ground not frozen (S_u_1, S_u_3). In the remaining two cases (S_u_2, S_u_4), changes in the concentration of K^+^ were so complex that an unambiguous classification as clockwise hystereses based on graphical analysis was not possible, despite this being the classification suggested by an analysis of regression residuals (Table 3, Fig. 8). Clockwise hysteresis for K^+^ concentrations in the agricultural catchment was produced for a 6-day flood event caused by long rainfall (R_p_3) of 110.6 mm and characterized by a mean intensity of 0.92 mm h^−1^ (Fig. 6). Clockwise hystereses in the studied agricultural catchment were also produced for abrupt floods caused by storms (R_s_2) characterized by precipitation of 40.4 mm and a mean intensity of 7.7 mm h^−1^ as well as preexisting high soil moisture levels (Fig. 6).

Both clockwise and anticlockwise K^+^ hystereses were produced for the studied mixed-use catchment for different types of flood events; however, clockwise hysteresis was more common. K^+^ hysteresis in stream draining the mixed-use catchment was quite complex for several flood events (R_s_2, R_p_2, S_u_2, S_u_4), which made it impossible to classify them as either clearly clockwise or clearly anticlockwise. This was due to significant variability in the K^+^ concentration, as measured during the studied flood waves. In addition, it is worth noting that the K^+^ hysteresis produced for the mixed-use and agricultural catchments were anticlockwise for rainfall floods occurring in catchments characterized by dry soil conditions (R_s_1; see Fig. 6, R_s_3). On the other hand, K^+^ hysteresis produced for rainfall floods occurring in catchments characterized by preexisting high soil moisture levels (R_s_2, R_p_3) were clockwise (Fig. 6).

## Discussion

### Relationship Between K^+^ Concentration and Discharge—Sources of Catchment K^+^

The present study has shown that K^+^ concentrations are characterized by a poor or statistically insignificant relationship with discharge relative to that with other main ions (Siwek et al. 2011). The reason for this pattern is the fact that K^+^ in the studied catchments comes from a variety of different sources reaching stream bed at different stages of the flood wave and characterizing by different and varying over time concentration of K^+^. Two main sources (paths of delivery) of K^+^ are overland flow and throughflow (areal sources). The mean K^+^ concentration of overland flow during a long-lasting rainfall event (July–August 2004) equaled 5.3 mg dm^−3^ (*n* = 6) in the agricultural catchment, and 3.8 mg dm^−3^ (*n* = 3) in the woodland catchment. During a snowmelt event with frozen soils (March 2003), the mean concentration of K^+^ in overland flow equaled 2.8 mg dm^−3^ (*n* = 15) in the agricultural catchment and 3.7 mg dm^−3^ (*n* = 7) in the woodland catchment. K^+^ delivery with overland flow is facilitated by a quite high quantity of clay fraction (< 0.002 mm) from the soils of the Stara Rzeka catchment; according to Barré et al. (2007, 2008), K^+^ ions are easily adsorbed by clay minerals. In surface soil horizons found on the slopes of the agricultural catchment of Kubaleniec, the clay fraction content ranges between 12 and 16% while the K^+^ exchangeable content ranges between 0.37 and 0.39 cmol_c_ kg^−1^ (unpublished data). It should be noted that loess-like deposits prevailing in the study area are very susceptible for erosion. Some quantity of K^+^ is also transported towards streams via throughflow affecting agricultural fields that utilize organic and inorganic fertilizer. The K^+^ concentration of throughflow varied depending on soil moisture antecedent conditions. The highest K^+^ concentration (38.6 mg dm^−3^) was observed in the agricultural catchment in an early stage of a storm event preceded by 18 days without rainfall, while with high soil moisture, the concentration decreased to 0.3 mg dm^−3^. In the woodland catchment, the throughflow K^+^ concentration ranged from 0.8 to 8.7 mg dm^−3^.

It is important to note that the mean amount of potassium fertilizer used in Małopolska Province, where the Stara Rzeka catchment is located, is less than half that used in European Union countries: 19.7 versus 40 kg year^−1^ (Barré et al. 2007; Central Statistical Office 2011). Some K^+^ is also released upon the shifting of streambed sediments and thanks to bank erosion during the rising limb of a flood wave. This is crucial in agricultural catchments where exchangeable K^+^ content in streambed and riverbank sediments is higher than in the woodland catchment (Table 4). It is the consequence of the higher delivery of K^+^ from the hillslopes as well as the higher content of clay fraction in sediments in agricultural catchments. In agricultural catchments such as Kubaleniec and Stara Rzeka, point sources of K^+^ are very important and include untreated wastewater that enters roadside ditches and later local streams. According to Pietrzak (2005), the quantity of untreated household wastewater released into local streams in the Stara Rzeka catchment equals 6 to 7 mm year^−1^. Hence, increased discharge in the course of flood events causes a decrease in K^+^ concentration thanks to the dilution of wastewater from point sources as well as an increase in K^+^ concentration due to the flushing of soils across areal sources and the delivery of K^+^ from these sources to streams via eroded soil particles.

**Table 4:** Average concentration of exchangeable K^+^ and particle-size distribution in streambed and riverbank sediments (n – number of samples)

Streams	Streambed sediments					Particle-size distribution	Riverbank sediments					Particle-size distribution
	*n*	K^+^	Sand	Silt	Clay		*n*	K^+^	Sand	Silt	Clay	
		cmol kg^−1^	%					cmol kg^−1^	%			
Leśny Potok (woodland)	3	0.12	53.3	42.0	4.7	Sandy loam	6	0.13	37.2	52.8	10.0	Silt loam
Kubaleniec (agricultural)	3	0.18	16.0	74.0	10.0	Silt loam	6	0.13	13.8	74.5	11.7	Silt loam
Dworski Potok (agricultural)	3	0.28	21.6	70.7	7.7	Silt loam	6	0.29	15.7	72.8	11.5	Silt loam
Stara Rzeka (mixed)	1	0.16	56.0	38.0	6.0	Sandy loam	2	0.17	31.5	56.0	12.5	Silt loam

Each source of K^+^ in the studied catchments releases K^+^ at different stages of the flood wave, which further serves to weaken the relationship between K^+^ concentration and discharge. This trend was observed for storm floods in the Kubaleniec agricultural catchment in July of 2002. A small flood event occurred on July 15th and 16th and was characterized by discharge reaching 0.6 dm^3^ s^−1^. The first high peak in K^+^ concentration in stream water was most likely caused by the shifting of streambed sediments and bank sediments, as evidenced by the simultaneous increase in suspended matter concentration (data not shown). The second peak lasted longer and was caused by the flushing of K^+^ out of the soils by precipitation and its delivery to the stream channel via throughflow. Another flood occurred on July 16th and 17th and was characterized by much larger discharge (up to 242 dm^3^ s^−1^). In this case, the first peak in K^+^ concentration was associated with the shifting of streambed sediments and bank erosion, while the second peak was caused by the influx of overland flow. The K^+^ concentration began to increase again once the flood wave began to recede, although the rate of increase was small due to the influx of throughflow from distant parts of the catchment. Sandén et al. (1997) observed two K^+^ concentration peaks during a storm flood in a stream draining a small woodland catchment in southeastern Sweden. The first K^+^ peak was attributed to potassium-rich bank and channel material in the studied stream. The second peak, occurring during the falling limb of the flood, was explained in terms of K^+^ flushing in the upper layers of the soil. Hyer et al. (2001) also observed two K^+^ concentration peaks during storm floods in a small agricultural catchment in the USA, known as Muddy Creek. The first peak was attributed to potassium-rich overland flow reaching the studied stream, while the second peak was attributed to the arrival of soil water.

### Effect of Land Use on Stream K^+^ Concentrations

Land use in the three studied catchments plays an important role in the concentration of K^+^ in stream water. K^+^ concentrations during flood events, as well as prior to flood events, were markedly lower in the woodland catchment than in agricultural and mixed-use catchments due to a lower supply of K^+^ in the woodland catchment. The lower K^+^ concentration in stream water in the woodland catchment in pre-flood periods is the result of lower K^+^ concentrations in groundwater. According to Żelazny (2005a), the mean K^+^ concentration in groundwater—well water—in the Silesian unit, the geological parent material of the woodland catchment, is 3.9 mg dm^−3^. The mean K^+^ concentration in groundwater in the Sub-Silesian I and II units, the main geological parent material of the agricultural and mixed-use catchments, is 8.2 and 27.7 mg dm^−3^, respectively. According to Żelazny (2005a) and Siwek (2012), human impact play an important role in the determining the K^+^ concentration in ground water of the Sub-Silesian I and II units. Important sources of K^+^ in groundwater in the agricultural and mixed-use catchments are leaking septic tanks as well as organic and mineral fertilizers. These two catchments do not possess sewer lines or wastewater treatment plants. Nowadays, it is very hard or even impossible to unambiguously distinguish the impact of geology from the impact of land use on the K^+^ concentration in ground water. The large supply of K^+^ in the agricultural and mixed-use catchments was the cause of a large peak in K^+^ concentration in stream water at the beginning of the studied flood events. This occurred via the shifting of streambed sediments rich in K^+^ as well as via soil flushing by rainfall and snowmelt. The K^+^ concentration in stream water in the agricultural catchments reached 10–11 mg dm^−3^ at the beginning of each studied flood event. The K^+^ concentration, for the same time period, in the woodland catchment reached only 3–4 mg dm^−3^. Foster (1978) investigated a small agricultural catchment in East Devon in the UK and found that seasonal changes in land use affected the K^+^ concentration in stream water during flood events. K^+^ concentrations were found to be lower during the crop growing period, as the amount of K^+^ washed away from the soil surface by high-intensity rainfall decreased. On the other hand, the plowing season produced increased K^+^ concentrations in stream water thanks to a high rate of K^+^ washout across plowed soil surfaces. However, our research work in agricultural and mixed-use catchments has not shown a relationship between seasonal work in the fields and a resulting change in land use and K^+^ concentrations in stream water during flood events.

Despite the large supply of K^+^ in the agricultural and mixed-use catchments, a gradual depletion is very likely under certain circumstances. This process was observed during a period of several subsequent snowmelt flood events. A similar process was observed by Holz (2010) in a stream draining a small agricultural catchment in Tasmania. In our research, we did not observe an increase in K^+^ in stream water at the end of the snowmelt season—a reference to K^+^ originating in the flushing out of decaying organic matter accumulated in snow during the winter, as described by Stottemyer (2001) who stated that higher air and snow temperatures favor such an increase towards the end of the snowmelt season. In the agricultural and mixed-use catchments we investigated, the accumulation of organic matter in the snow cover was limited due to its complete disappearance during selected snowmelt events. Accumulation was possible in the studied woodland catchment where the snow cover did not disappear altogether in the course of subsequent snowmelt events. However, despite the observed higher concentrations of K^+^ during the second snowmelt flood in March of 2013, compared with the first studied snowmelt flood in January of 2013, the small number of flood events studied makes it impossible to draw any solid conclusions.

### Effect of Seasonality and Flood Type on K^+^ Concentration in Stream Water

Our research has shown that flood type determines the K^+^ concentration in stream water in the course of flood events. This relationship has not been published on extensively, as most papers tend to focus on rainfall floods (Foster 1978; Sandén 1997; Holloway and Dahlgren 2001; Holz 2010). Higher concentrations of stream water K^+^ in the woodland catchment during mid-winter and spring snowmelt floods versus summer rainfall floods indicate that the seasonal (vegetation) factor, i.e., a larger intake of K^+^ by plants in the summer, plays an important role in determining stream water K^+^ concentration in the woodland catchment during floods. The influence of vegetation cover was not observed in the agricultural and mixed-use catchments, where higher concentrations of stream water K^+^ were noted during summer rainfall floods versus snowmelt floods. Our research has shown that in the agricultural and mixed-use catchments, featuring a very large pool of K^+^, a much more important factor than the plant potassium intake was the ability to flush K^+^ out of the soil. The strongest likelihood of K^+^ being flushed out of the soils in agricultural and mixed-use catchments occurs during summer rainfall floods, both storm floods and long rainfall floods. This was due to good rainwater infiltration conditions, which produced good conditions for flushing K^+^ out of the soil. The infiltration of snowmelt water in the soil was limited during snowmelt floods, especially floods with the ground frozen, which made it less possible to flush out K^+^. This relationship was also confirmed by Siwek et al. (2013a) using factor analysis that showed that the higher the soil temperature at a depth of 5 cm, the higher the K^+^ concentration in stream water in agricultural and mixed-use catchments. Opposite patterns were noted for the woodland catchment, where a seasonal factor was identified (Siwek et al. 2013a).

### Role of Water Supply Patterns and Antecedent Conditions on the K^+^/Q Hysteresis Pattern

The hysteresis effect was observed to describe changes in the K^+^ concentration in the studied streams during flood events. The existence of this effect in the case of K^+^ was also documented by Holz (2010) as well as Evans and Davies (1998). Our research has shown that the concentration of K^+^, at a given specific runoff, in the course of virtually all flood events in the woodland catchment was higher during the rising limb versus the falling limb of the flood wave. The one exception consisted of a snowmelt flood with the ground not frozen. This trend was determined by the studied streams’ water recharge mechanisms and the supply of K^+^ in each given catchment. Stream flow during flood events occurring in the woodland catchment was mostly determined by two components of runoff—groundwater and throughflow. The significance of overland flow was marginal. A dense network of various types of gullies cutting into slopes in the woodland catchment played a special role in flow patterns associated with throughflow and its path to stream channels during flood events. Precipitation water infiltrating the soils was drained quite rapidly and would accelerate upon reaching the surface. According to Dunne and Black (1970), the rate of acceleration ranged between 100 and 150 times the flow rate in the soils. Clockwise K^+^ hystereses for a stream draining the woodland catchment indicate flushing and a gradual loss of K^+^ from the soils in the course of single flood events. Our research confirmed the findings of Evans and Davies (1998) in that soil flushing may lead to the formation of clockwise convex hystereses for streams recharged during floods by two components of runoff. No distinct increase in K^+^ concentration was noted in the early stages of the increase in discharge in the woodland catchment in the case of all but one flood—a long rainfall flood. This type of increase would suggest the delivery of K^+^ to the studied stream channel along with shifting streambed material and bank material. The evidence for this was low exchangeable K^+^ content in sediments accumulated in the channel of the stream draining the woodland catchment (Table 4). Most of the sediment consisted of sand whose K^+^ adsorption capabilities are quite poor (Monger and Kelly 2002).

Clockwise K^+^ hystereses in the agricultural catchment were noted for storm-driven floods and floods produced by long rainfall of high intensity. Higher K^+^ concentrations in the rising limb versus the falling limb of the studied flood wave were produced by (i) the shifting of accumulated streambed sediments and bank sediments, (ii) delivery of K^+^ as part of overland flow, and (iii) delivery of K^+^ via throughflow. The delivery of K^+^ via shifting streambed sediments and bank sediments was inferred from a rapid and substantial increase in K^+^ concentration at the beginning of the flood wave. Silt loam or sandy loam particle-size distribution of material that lines the streambed in the agricultural catchment is undoubtedly good at adsorbing and storing K^+^ arriving from various sources including untreated wastewater from area farms. Overland flow played an important role in the delivery of K^+^ to the stream draining the agricultural catchment. It intensified rapidly during floods caused by rainfall of high amount and high intensity, which was confirmed via measurements of the concentration of PO_4_^3−^ and the measurement of specific conductivity (Siwek et al. 2009, 2013b). Overland flow was most likely to form along dirt roads, paths, and rills separating agricultural fields. It also tended to form relatively rapidly on farmland found on hill slopes following soil saturation with water and a decrease in the rate of infiltration. The rapid formation of overland flow was also facilitated by the shallow presence of a clayey argillic (fragipan) horizon, which created a barrier halting infiltrating water (Szymański and Skiba 2013). The argillic horizon was often encountered at the surface due to the erosion of the upper soil layers facilitated by the plowing of farm fields. Only throughflow and groundwater contributed K^+^ ions to the stream draining the studied agricultural catchment during the falling limb of the flood wave. As the rain ceased, overland flow disappeared very quickly from the slopes.

Overland flow in the agricultural catchment of Kubaleniec appeared following soil saturation during long-lasting floods caused by continuous, low-intensity rainfall. This explains why the K^+^ concentration was higher during the falling limb versus the rising limb of the flood resulting in anticlockwise hystereses. A similar situation was observed during snowmelt floods occurring with the ground not frozen, which was able to saturate with at least some of the water from melting snow in the early stages of each studied flood event. Anticlockwise hystereses were also produced for snowmelt floods with the ground frozen; however, the formation mechanism for these hystereses was different from that for floods with the ground not frozen. Water from melting snow flowing on the frozen ground had a significant contributor to the stream at the beginning of each given snowmelt flood. The snow cover was characterized by low K^+^ concentrations, as shown by the research of Żelazny (2005b) who found that the average concentration of K^+^ in winter precipitation was 0.2 mg dm^−3^ in the period 2002–2004. One significant moment in this type of flood is the incision made by snowmelt water in the unfreezing ground. This leads to an increase in the K^+^ concentration both in snowmelt water flowing across the catchment surface as well as in stream water (Fig. 6c). The flushing out of K^+^ from an increasingly thick layer of unfreezing soil leads to a higher K^+^ concentration during the falling limb of the flood wave (versus the rising limb). According to Evans and Davies (1998), hysteresis direction and width are determined by the concentration of ions in each given runoff component, given a constant sequence of runoff components in each studied catchment. Our hydrochemical research and fieldwork indicate that the sequence of the arrival of each runoff component is not constant per catchment and the sequence may change depending on flood type and hydrometeorological conditions in each given catchment affected by a flood event.

The size of the catchment was an additional factor modifying the K^+^ supply pattern; however, this effect was partially connected with differences in land use in the bigger catchment. The studied mixed-use catchment was characterized by variable arrival times for water coming in from various parts of the catchment, with each part producing water with a different concentration of K^+^. The main type of K^+^ hysteresis was clockwise, which was associated with the faster arrival of water rich in K^+^ at the terminus of the studied catchment. This water rich in K^+^ originated in a nearby agricultural part of the mixed-use catchment. Water from the more distant woodland part, characterized by a smaller supply of K^+^, was detected at the terminus of the mixed-use catchment following a time delay.

K^+^ hysteresis are much narrower in the woodland catchment than in the studied agricultural catchments due to smaller differences in K^+^ concentration noted in the various runoff components in the woodland catchment. In the studied agricultural and mixed-use catchments, the broadest hysteresis loops were noted for summer rainfall floods occurring under dry catchment conditions. This is due to the large pool of K^+^ stored in the soils during periods without rainfall occurring prior to flood events and strong ability of throughflow flushing K^+^ out of the soils. Evans and Davies (1998) observed seasonal changes in hysteresis width, which they attributed to changing K^+^ concentrations in soil water over the course of the year. In our research, K^+^ hysteresis width was determined not only by the pool of K^+^ in the catchment immediately prior to a flood event but also by the ability of its delivery to a stream channel. This ability increases with increasing precipitation volume and intensity responsible for a given flood event.

## Conclusions

Our research has shown that land use, hydrometeorological conditions, and seasonality play an important role in determining K^+^ concentrations in stream water during flood events. These factors determine the pool of K^+^ available for transport via infiltrating water and affect the water recharge mechanisms in action during flood events. In the woodland catchment, seasonality plays an important role in determining stream water K^+^ concentration during floods. Intake of K^+^ by plants in the summer results in a lower concentration of stream water K^+^ during summer rainfall events than during mid-winter and spring snowmelt events. In the agricultural and mixed-use catchments, the influence of seasonality on stream water K^+^ concentration during floods was not observed. Agricultural and mixed-use catchments possess a larger pool of K^+^ than woodland catchments, which results in higher K^+^ content in stream water during flood events. The pool of K^+^ depends on soil moisture conditions in each given catchment immediately prior to a flood event. The pool of K^+^ is larger during floods preceded by a long period without rainfall that results in very dry catchment conditions versus floods preceded by periods high in soil moisture. The larger the pool of K^+^ in the catchment, the larger the difference in K^+^ concentration during the rising and falling limbs of the flood wave, which results in a wider K^+^ hysteresis loop.

Catchment land use and hydrometeorological conditions characteristic of flood types such as storm floods and long rainfall floods as well as snowmelt floods with the ground frozen and not frozen are instrumental in the manner of recharging streams with water. The recharging mechanism is vital in the process of flushing out K^+^ from the soils and its delivery to stream channels along with groundwater, overland flow, and throughflow. Higher K^+^ concentrations in stream water in each studied agricultural and mixed-use catchment were recorded in the course of summer rainfall floods versus snowmelt floods—at a given specific runoff. In all studied catchments, the lowest K^+^ concentrations were recorded during snowmelt floods with the ground frozen.

**Fig. 9:**
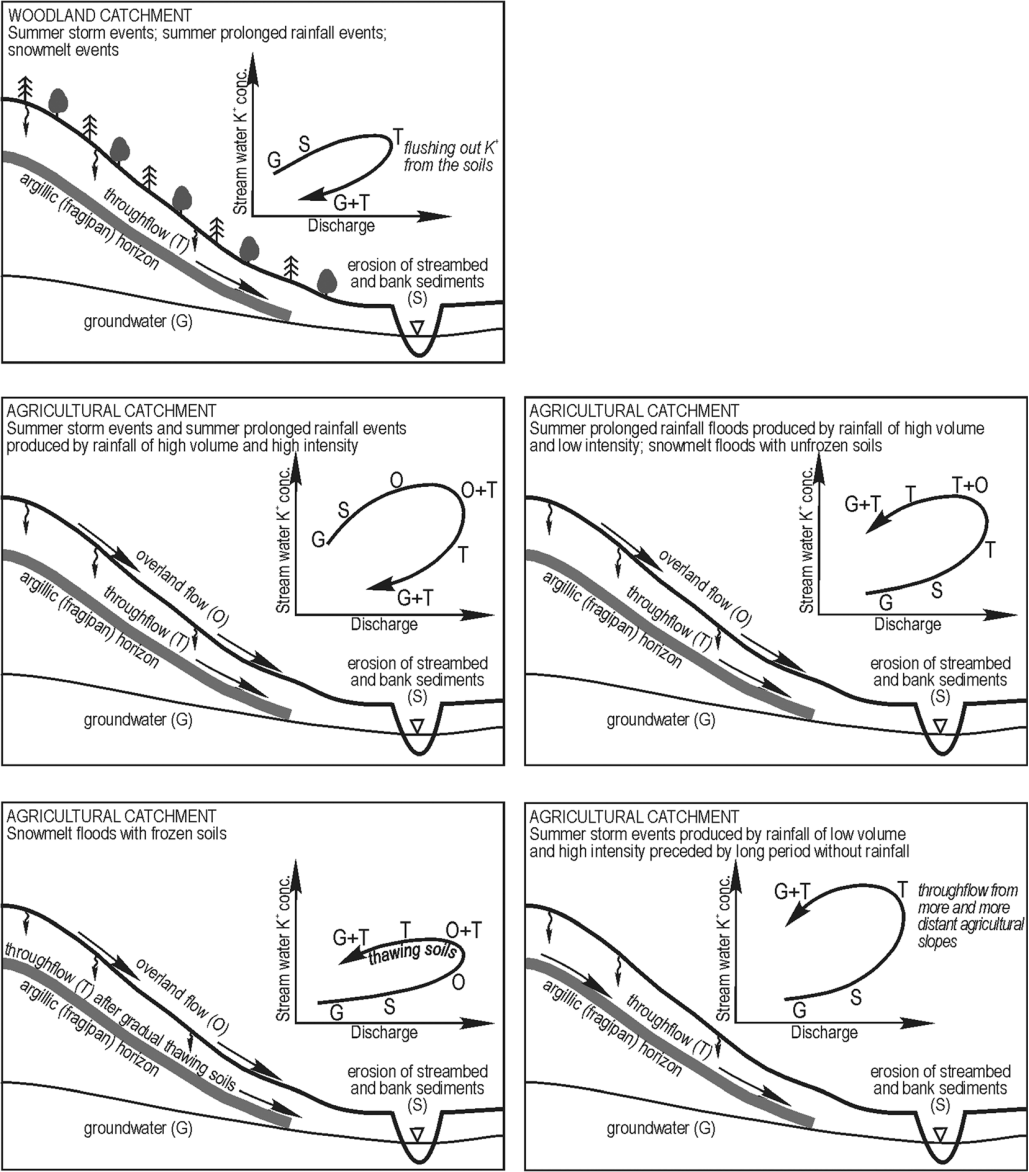
Scheme of main sources and pathways of K^+^ in stream water during floods of different types in woodland and agricultural catchments

The sequence of runoff components was not fixed in each given catchment and depended on hydrometeorological conditions in each studied catchment in the course of each flood event and immediately prior to it. The direction of K^+^ hysteresis in the course of floods was determined by the time it takes for overland flow and throughflow to reach stream channels (Fig. 9). Clockwise hysteresis was produced for all flood types in the woodland catchment except for snowmelt floods with the ground not frozen. This hysteresis direction was associated with the flushing out of K^+^ from the soils and its delivery via throughflow. Overland flow played a marginal role in the woodland catchment. In the agricultural catchment, clockwise hystereses were produced for high-intensity, high-volume rainfall floods. This type of strong precipitation made it possible for rapid formation of overland flow, which served as a key source of K^+^ in stream water. Anticlockwise hystereses were produced for the agricultural catchment for low-intensity rainfall floods as well as for snowmelt floods with the ground frozen and not frozen. In the mixed-use catchment, the direction of K^+^ hysteresis depended additionally on the time it takes for water to reach the main stream from different parts of the catchment characterized by different land use. Table 5 contains the main findings of the study.

**Table 5:** Summary of main findings

	Role of land use	Role of seasonality and hydrometeorological conditions
Concentration of K^+^	Higher K^+^ concentration in the streams draining agricultural and mixed-use catchments than in the stream draining woodland catchment	In the streams draining agricultural and mixed-use catchments, higher K^+^ concentration during summer rainfall floods than during snowmelt events. In the stream draining woodland catchment, lower K^+^ occurred during summer floods than during snowmelt floods. In all streams, lower K^+^ concentrations occurred during snowmelts with the ground frozen than during snowmelts with the ground unfrozen
K^+^ hysteresis size	Wider K^+^ hystereses in the streams draining agricultural and mixed-use catchments than in the stream draining woodland catchment	Wider K^+^ hystereses during summer rainfall floods than during snowmelt floods. In the case of summer rainfall floods, wider K^+^ hystereses during floods preceded by a long period without rainfall (low soil moisture levels) versus floods preceded by period with rainfall (high soil moisture levels)
K^+^ hysteresis direction	In the stream draining woodland catchment: clockwise K^+^ hystereses for all flood types, except for snowmelt floods with the ground not frozen. In the stream draining agricultural catchment: clockwise hystereses for short- and long-duration rainfall floods caused by high-intensity, high-volume rainfall; counterclockwise hystereses for short and long duration rainfall floods caused by low-intensity, low-volume rainfall as well as during snowmelt floods with the soil frozen and not frozen. In the mixed-use catchment: the hysteresis direction was affected by different lag times for water reaching stream channels from areas with different land use	
